# Molecular remission using low-dose immunotherapy for relapsed refractory Philadelphia chromosome-positive precursor B-cell acute lymphoblastic leukemia post-allogeneic stem cell transplant

**DOI:** 10.2144/fsoa-2019-0009

**Published:** 2019-04-12

**Authors:** Dipnarine Maharaj, Pedro Vianna, Gabriel DeCarvalho, Delaram Pourkalbassi, Christopher Hickey, Jacqueline Gouvea

**Affiliations:** 1 The Maharaj Institute of Immune Regenerative Medicine, Boynton Beach, FL 33437, USA; 2 Stanford University Department of Medicine, Stanford, CA 94305, USA; 3 Florida State University College of Medicine, Tallahassee, FL 32306-4300, USA

**Keywords:** allo-HCT, immunotherapy, natural killer cell, refractory Ph^+ ^ALL, rIL-2

## Abstract

Adults with relapsed/refractory acute lymphoblastic leukemia have a poor prognosis. While current immunotherapies are promising, they are toxic, with graft-versus-host disease a major complication of allogeneic therapy. Here, we report a patient with high-risk relapsed/refractory Philadelphia chromosome-positive B-cell acute lymphoblastic leukemia (ALL) following chemotherapy induction, matched related donor allogeneic hematopoietic stem cell transplantation (allo-HCT), donor lymphocyte infusion and two tyrosine kinase inhibitors. The patient achieved a complete molecular and cytogenetic remission with minimal adverse events or evidence of GVHD following recombinant human IL-2 (rIL-2), in combination with a tyrosine kinase inhibitor (TKI). There was a ninefold increase in natural killer (NK) cell activity and natural killer T cells (NKT) cells (CD2^+^CD26^+^). Personalized low dose recombinant human IL-2-mediated NK cell stimulation represents an effective, nontoxic immunotherapy administered in the outpatient setting for relapsed acute lymphoblastic leukemia and warrants further investigation.

## Lay abstract

Recurrent or refractory adult acute lymphoblastic leukemia (ALL) is often associated with dismal outcomes after standard treatment. Despite great progress in recent immunotherapy approaches, ALL patients encounter a therapeutic challenge due to the toxicity and complications of these new treatments. We report a patient with high-risk relapsed/refractory Philadelphia chromosome-positive B-cell ALL following standard treatment and in whom a personalized immunotherapy treatment resulted in molecular remission with minimal side effects and maintenance of immune tolerance. This nontoxic treatment approach can be used to treat patients with relapsed/refractory ALL and needs to be evaluated in future research.

## Graphical abstract

Personalized low-dose recombinant human IL-2 (rIL-2)-mediated natural killer (NK) cell stimulation, in combination with a tyrosine kinase inhibitor (TKI), resulted in activation of effector NK cells and an increase in IFN-γ and (T cells + NK cells expressing dipeptidyl peptidase) levels. The CD4^+^CD25^+^ regulatory T cells (Tregs) showed a progressive decrease. This personalized treatment approach which targeted Ph+ acute lymphoblastic leukemia t (9; 22) resulted in molecular remission with minimal side effects.

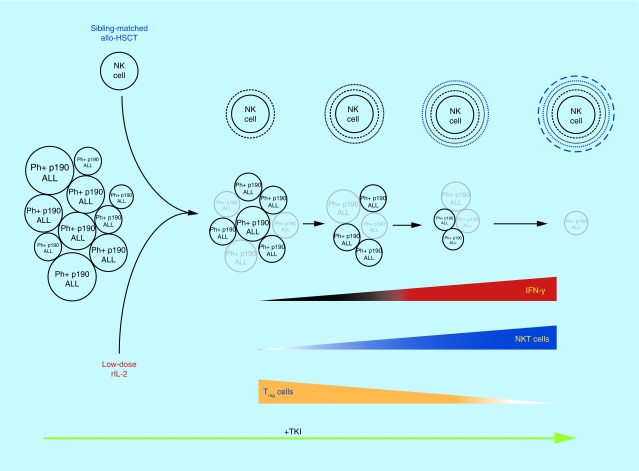

Adult patients with refractory acute lymphoblastic leukemia (ALL) or who relapse after allogeneic hematopoietic stem cell transplantation (allo-HCT) have dismal outcomes with a median survival of less than 1 year; less than a quarter of patients survive 3 years [1].

Therapeutic options for salvage remission induction post-HCT in ALL patients currently include various immunotherapeutic approaches including donor lymphocyte infusions (DLI), blinatumomab, inotuzumab ozogamicin (InO) or chimeric antigen receptor T cells (CAR-T) as well as conventional cytarabine-based chemotherapy regimens, a second allo-HCT, participation in a clinical trial or supportive care [2]. In some cases, remissions can be induced solely by restoring graft-versus-tumor activity with DLI or with withdrawal of immune suppression alone [3]. Other agents, such as InO, an anti-CD22 monoclonal antibody–drug conjugate [2], and blinatumomab, a bispecific T cell engager monoclonal antibody directed at CD19 on precursor B-cell ALL and CD3 on cytotoxic T cells, have shown superior complete remission (CR), overall survival and progression free survival rates compared with conventional chemotherapy regimens [2,4]. Tisagenlecleucel, a CD19-directed chimeric antigen receptor autologous T cell immunotherapy, has shown up to 83% overall remission in children and young adults with precursor B-cell ALL. However, these novel antibody and cell-mediated-based immunotherapies are not without severe adverse events. DLI post-HCT is associated with graft-versus-host disease (GVHD) in 60–70% of patients and bone marrow hypoplasia in 20–40% [5,6]. InO has been linked to veno-occlusive disease in 11% of patients who did not go on to receive HCT and up to 22% of patients who did go on to receive HCT^2^, limiting its use in patients post allo-HCT. Blinatumumab and tisagenlecleucel can cause cytokine release syndrome (CRS) and neurotoxicity, each of which may be life-threatening or fatal, and thus require hospitalization around time of initial infusion [7].

Despite the advances in allogeneic immunotherapy, immune escape (i.e., tumor evasion of the donor immune system) likely plays a fundamental role in the pathogenesis of relapse. Immune escape can be mediated by high levels of checkpoint receptors, such as CTLA-4 and PD-1, on donor-derived lymphocytes or high expression of their cognate ligands on residual tumor cells. For patients who relapse after allo-HCT, immune checkpoint inhibition has led to high response rates in lymphoma patients but with significant immune breakthrough GVHD with mortality rates of 25% [8].

As a result, while allogeneic immunotherapy remains promising, there remains no clear-cut first-line agent to address relapse after transplantation and we continue to search for means of inducing antitumor immunity while mitigating the risk of GVHD and limiting toxicity.

Natural killer (NK) cells are highly cytotoxic innate effector lymphocytes capable of killing their targets rapidly, broadly and in a nonspecific manner and have shown cytotoxicity against a number of hematologic malignancies including ALL [9–12]. Their inability to cause GVHD and their potent graft versus tumor effects in allo-HCT makes NK cells uniquely primed for use in immunotherapy [13–15]. IL-2, a cytokine and activator of NK and cytotoxic T cell function, has been shown to have an antitumor effect including recipients of syngeneic bone marrow transplant [16]. IL-2 stimulates NK cell proliferation, cytotoxic activity and cytokine production but has also been shown to stimulate regulatory T cells (Tregs) [17].

Therapeutic uses of IL-2 have utilized two different strategies by varying the dose of IL-2; ultralow doses to reduce autoimmune responses through Treg stimulation and low doses to augment NK cell-mediated antitumor immune responses [18]. This dualistic pro and anti-immune function of IL-2 based on dosing and frequency can be utilized in a personalized therapeutic approach in immune-oncology in which NK cell antitumor responses can be augmented while modifying Treg activity to limit GVHD.

In this case report, we describe a middle-aged male patient presenting with high-risk relapsed Philadelphia chromosome-positive (Ph^+^) B-cell ALL following a matched related donor allogeneic HCT, donor lymphocyte infusion and two tyrosine kinase inhibitors (TKIs). The patient achieved a complete molecular and cytogenetic remission following four cycles of low-dose recombinant human IL-2 (rIL-2) in combination with a TKI with minimal adverse events.

## Case report

A 39-year-old man attended our clinic in March 2017 with a two-year history of Ph^+^ precursor B-cell ALL. In June 2015 he had attended an emergency department with a 2-week history of fatigue, lethargy, backache, leg and rib pain refractory to opioids. Blood counts revealed a leukocytosis with white cell count of 67 × 10^9^ cells/l with 34% lymphoblasts measured by flow cytometry. Hemoglobin was 12.3 g/dl and platelet count at 49 × 10^9^/l. Bone marrow aspirate/biopsy showed a precursor B-cell ALL, Ph^+^ (t 9; 22). The patient was enrolled in the UKALL14, version 6 Protocol (NCT01085617: ClinicalTrials.gov) which included a five-drug induction regimen in adults with *de novo* ALL between 25 and 65 years. Induction Phase I: PEG-ASP (1000 IU/m^2^) on days (d) 4 and 18, daunorubicin 30 mg/m^2^ and vincristine 1.4 mg/m^2^ on d1,8,15 and 22, dexamethasone 10 mg/m^2^ d1–4, 8–11,15–18 and intrathecal methotrexate (ITMTX) 12.5 mg on d14. Patient received continuous imatinib 400 mg escalating to 600 mg daily throughout induction treatment. Phase II induction: cyclophosphamide 1000 mg/m^2^ d1,15, Ara-C 75 mg/m^2^ d2–5, 9–12, 16–19 + 23–26, mercaptopurine 60 mg/m^2^ throughout and intrathecal methotrexate d1, 8, 15, 22.

After Phase II induction the patient achieved a complete molecular remission with negative *BCR-ABL1* p190 transcripts by reverse transcription polymerase chain reaction (RT-PCR). Complications included constipation, febrile neutropenia and pneumonia. He was consolidated with myeloablative conditioning including cyclophosphamide/total body irradiation (TBI), followed by a matched (brother) allogeneic stem cell transplant. Post-transplant he developed hyponatremia, total body irradiation somnolence and grade IV acute cutaneous GVHD managed with steroids. Post-transplant maintenance included imatinib, infection prophylaxis and GVHD prophylaxis with methotrexate and cyclosporine. Disease relapse occurred at 6 months post-transplant with *BCR-ABL1* p190 transcript expression at a level of 18.23%, confirmed on bone marrow biopsy at 9 months post allo-HCT. Imatinib was replaced by the TKI dasatinib 140 mg daily, and followed by DLI (3 × 10^6^ CD3 cells/kg), resulting in second remission with negative minimal residual disease (MRD) by flow cytometry. Three months later, disease progression occurred with expression for *BCR-ABL1* p190 in 163275 copies of control gene. He was given an overall life expectancy of less than 1 year.

The patient attended our clinic for evaluation and treatment. Options discussed included responses and side effects of chimeric antigen receptor T cells cell therapy, antibody–drug conjugates, such as InO or blinatumomab, chemotherapy, a second HCT and/or DLI, no therapy and palliative care or experimental personalized low-dose immunotherapy. The patient gave informed consent for the experimental immunotherapy including publication of results.

The treatment consisted of daily low-dose subcutaneous rIL-2 with variation of dosing and frequency of administration based on measurement of an extensive peripheral blood immune panel including NK cells, NK cell cytotoxicity, B cells, T cells and Treg cells to selectively stimulate a graft-versus-leukemia response while minimizing GVHD. Simultaneously, cytokine levels including IFNγ in plasma were measured. The patient received a total of four cycles (4–7 weeks) of rIL-2 injections of 10–20,000 IU/kg, 5 days per week. The daily dose and duration of each cycle was based on the results of the peripheral blood immune panels. Cycles 1 and 2 were 6 weeks, cycle 3 was 7 weeks and cycle 4 was 4 weeks. Dasatinib was continued at 140 mg daily. Expression of *BCR-ABL1* p190 transcript was monitored intermittently using RT-PCR throughout his rIL-2 treatment cycles.

**Figure 1.**
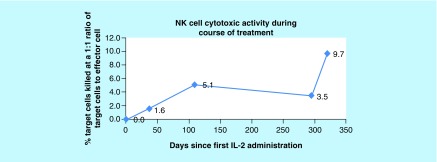
**Normal mean in healthy adults: 28.1% ± 11.8. From left to right, values reflect the cytotoxic activity of natural killer cells measured prior to the first treatment cycle (baseline), 40 days after the baseline, 109 days after the baseline, 294 days the after baseline and 320 days after the baseline (at the end of the fourth treatment cycle).** NK cell activity improved from 0.00% prior to initiation of cycle 1 of recombinant human interleukin-2 to 5.1% at the end of cycle 3 and to 9.7% by the end of cycle 4, representing a ninefold increase. In this assay the percent cytotoxic activity of NK cells in blood was measured by the release of 51Cr from NK cell-sensitive tumor cell targets (K562) following a 4h incubation of target cells with effector cells. NK: Natural killer.

**Figure 2.**
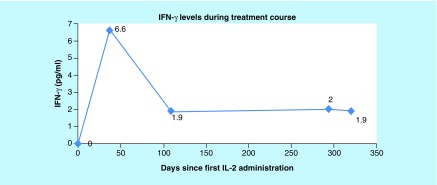
**Normal reference range 0.0–3.0 pg/ml.** From left to right, values reflect the IFNγ levels measured prior to the first treatment cycle (baseline), 40 days after the baseline, 109 days after the baseline, 294 days the after baseline and 320 days after the baseline (at the end of the fourth treatment cycle). IFN-γ level increased from 0.0 pg/ml prior to cycle 1, peaked at 6.6 pg/ml at the end of cycle1 and remained elevated through cycles 2, 3 and 4 with a level of 1.9 pg/ml at the end of the cycle 4. To determine the level of IFNγ, morning fasting blood samples were collected into EDTA anticoagulant tubes. Plasma was separated within 2 h of collection and stored at -80°C until assayed. The cytokine level in plasma was measured using Q-PlexTM Human Custom Cytokine – multiplex (18 plex). The multiplex assay is a quantitative chemiluminescent assay (ELISA) allowing concurrent measurement in samples where 18 distinct capture antibodies have been absorbed to each well of a 96-well plate in a defined array.

**Figure 3.**
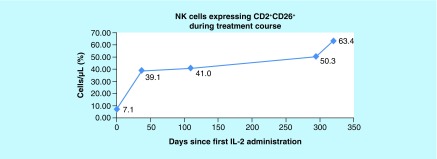
**Normal reference ranges 38–60%.** From left to right, values reflect CD2^+^CD26^+^ expressing NK cells measured prior to the first treatment cycle (baseline), 40 days after the baseline, 109 days after the baseline, 294 days the after baseline and 320 days after the baseline (at the end of the fourth treatment cycle). CD2^+^CD26^+^ (T cells + NK cells expressing dipeptidyl peptidase) increased from 7.1% prior to initiation of cycle 1 of recombinant human interleukin-2 to 63.4% at the end of the cycle 4, showing a ninefold increase. The number of NK cells in the blood was determined using the flow cytometer to measure the lymphocytes that were CD2^+^ CD26^+ ^and CD3^+^ and the complete blood count to determine the number of total lymphocytes for calculation of percent CD2^+^ CD26^+^ cells. NK: Natural killer.

**Figure 4.**
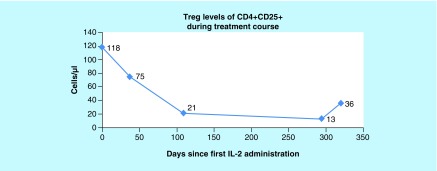
**Normal reference range 28–149 cells/µl.** From left to right, values reflect Tregs measured prior to the first treatment cycle (baseline), 40 days after the baseline, 109 days after the baseline, 294 days the after baseline and 320 days after the baseline (at the end of the fourth treatment cycle). The CD4^+^CD25^+^ Tregs showed a progressive decrease from the upper end to the lower end of the normal range. The number of Tregs in the blood was determined using the flow cytometer to measure the percentage of lymphocytes that were CD4^+^ CD25^+^ and the complete blood count to determine the number of lymphocytes. Treg: T regulatory cell.

**Figure 5.**
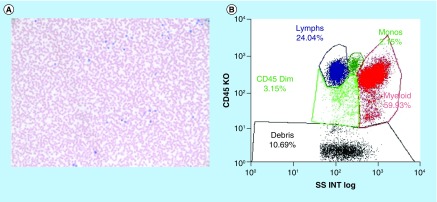
**Framework synoptic summation.** **(A)** Morphology – peripheral blood: anemia 10.5 g/dl with MCV 105.8 fl, no increased blasts or lymphoblasts. **(B)** Flow cytometry – no immunophenotypic abnormalities; no evidence of monoclonal lymphoid neoplasia; no evidence of increased myeloid or lymphoid blasts; plasma cells not increased. FISH study: all panel – no clonal abnormalities detected in the genomic regions analyzed. Cytogenetics – 46, XY;  interpretation: normal karyotype (male), showing no clonal abnormalities. Molecular: *BCR-ABL1* quantitative RT-PCR – *BCR-ABL 1* p210 – Not detected; *BCR-ABL 1* p190 – Not detected. MCV: Mean corpuscular volume; RT-PCR: Reverse transcription polymerase chain reaction; XY: Chromosomes.

Results showed NK cell activity improvement from 0% prior to initiation of cycle 1 of rIL-2 to 5.08% at the end of cycle 3 and to 9.68% by the end of cycle 4 (Figure 1). The CD56brightCD3^-^NKcells were high prior to starting rIL-2 and remained in the upper normal range throughout treatment. IFN-γ increased from 0.0 pg/ml prior to cycle 1, peaked at 6.6 pg/ml at the end of cycle 1 and remained elevated through cycles 2, 3 and 4 with a level of 1.9 pg/ml at the end of the cycle 4 (Figure 2). CD2^+^CD26^+^ (T cells + NK cells expressing dipeptidyl peptidase) increased from 7.1% prior to initiation of cycle 1 of rIL-2 to 63.4% at the end of the cycle 4 (Figure 3). The CD4^+^CD25^+^ Tregs which were at the upper end of the normal range showed a progressive decrease to the lower end (Figure 4). After cycle 2 bone marrow showed no abnormal lymphoid cells by flow cytometry, normal cytogenetics and no detectable levels of *BCR-ABL*, p190. Following cycle 3 peripheral blood showed no detectable levels of *BCR-ABL*, p190 or p210 transcripts (Figure 5), consistent with a complete molecular and cytogenetic remission with recombinant IL2 and a TKI. Immunotherapy with rIL-2 and TKI was well-tolerated; the only side effect was erythema (Grade1) at the injection site, which cleared rapidly after rIL-2 stopped. Dasatinib was continued at 140 mg daily. *BCR-ABL1* p190 transcript. Tregs, CD56brightCD3^-^NK cells and NK cell activity were not repeated since the patient returned to his home country. 21 months after starting rIL-2 the patient is well, asymptomatic and he has a normal quality of life. Notably, he successfully completed a triathlon.

## Discussion

We report a case of complete molecular and cytogenetic remission with personalized low-dose immunotherapy treatment using rIL-2 to increase NK cell function and natural killer T cells (NKT) levels in a patient with a poor prognosis high-risk relapsed/refractory Ph^+^ ALL (*BCR*-*ABL* p190) following allo-HCT. At the time of initiation of the salvage low dose IL-2, the patient had received the standard of care with combination TKI and induction chemotherapy followed by a matched sibling allogenic bone marrow transplant after achieving a CR, and subsequent DLI and additional TKI at first sign of minimal residual disease.

On the basis of extensive experimental data in animal models and results of treatment and prevention of relapse post allo-hematopoietic stem cell transplantation (HSCT) [19–22], we personalized the dose and schedule of low-dose IL-2 by monitoring immune effector cell levels and activity in the peripheral blood before and after treatment cycles (Table 1). There was a gap after each treatment cycle in order to minimize or prevent GVHD and IL-2-related side effects while increasing the activity of the donor immune effector cells against the Ph^+^ leukemic cell burden. The intention of allogeneic transplants and subsequent DLI is to utilize the donor's immune effector cells to target and eliminate the Ph^+^ leukemic cells as consolidative therapy. However, at the time of the patient's second relapse, the donor's NK cell activity was absent and reduced NKT cells expressing CD2^+^CD26^+^ were observed. However, following treatment with low-dose rIL-2, we observed increases in NK cell function and NKT cell numbers (Figures 1 & 3) which correlated with a CR as measured by *BCR-ABL1* p190 transcript analysis. NKT cells express dipeptidyl peptidase, which is an antigenic enzyme expressed on the surface of T lymphocytes and its increased surface expression after IL-2 has been reported to demonstrate tumor suppressing effects [23]. The increase in effector immune cell response was achieved with minimal side effects and no evidence of GVHD.

**Table 1.** **Personalized low-dose recombinant human IL-2 treatment.**

**Treatment course**	**Treatment period**	**End of cycle (# of days after baseline)**	**Treatment dose (million IU)**	**NK cytotoxic activity %**	**IFNγ (pg/ml)**	**CD2^+^CD26^+^ NKT cells (%cells/µl)**	**Treg CD4^+^CD25^+^ (%cells/µl)**
Baseline	–	–	–	0	0	7.1	118

Cycle 1	6 weeks	40	54	1.6	6.6	39.1	75

Cycle 2	6 weeks	109	54	5.1	1.9	41	21

Cycle 3	7 weeks	208	63	3.5	2	50.3	13

Cycle 4	4 weeks	320	36	9.7	1.9	63.4	36

Responses are from patient identified after each course of LD IL-2 therapy.

Treatment course consists of a period of treatment followed by a period of rest (no treatment) that was repeated on a personalized schedule as follows: cycle 1 treatment was given for 6 weeks followed by 30 days of rest. Cycle 2 treatment was given for 6 weeks followed by 53 days of rest. Cycle 3 treatment was given for 7 weeks followed by 86 days of rest and cycle 4 treatment was given for 4 weeks. Peripheral blood immune levels measured prior to the first treatment cycle (baseline), 40 days after the baseline, 109 days after the baseline, 294 days after the baseline and 320 days after the baseline (at the end of the fourth treatment cycle). NK cell cytotoxic activity showed a ninefold increase during the treatment course. IFN-γ increased with a peak at the end of cycle1 and remained elevated through cycles 2, 3 and 4. CD2^+^CD26^+^ (T cells + NK cells expressing dipeptidyl peptidase) increased during the course of treatment. The CD4^+^CD25^+^ Tregs which were at the upper end of the normal range showed a progressive decrease to the lower end.

LD: Low dose; NK: Natural killer; Treg: Regulatory T cell.

Although IL-2 has potent NK and T cell activity, recent studies have demonstrated that IL-2 is also critical for the establishment and maintenance of immune tolerance *in vivo* [21,24,25]. Saturation of high-affinity IL-2 receptors by low-dose IL-2 in chronic myelogenous leukemia (CML) patients after allo-HSCT and patients with cancers resulted in the *in vivo* selective expansion of CD56brightCD3^-^NK cells as well as CD4^+^CD25^+^ Tregs in peripheral blood, which results in dissecting GVT from GVHD effects [22,26]. In our patient, the CD56brightCD3^-^NK cell numbers were high but with no detectable cytotoxic function prior to starting low dose IL-2 and remained in the high normal range throughout treatment as the cytotoxic activity increased. It is believed that the decreased NK cell activity from 5.08 to 3.48% was due to a greater gap in treatment between the 3rd and 4th cycles following which the activity increased to 9.48%. We attribute the effectiveness of the low dose immunotherapy in this patient to the steady expansion in the NK cell functional activity observed, illustrating the importance of measuring the functional activity of immune effectors. The patient's CD4^+^CD25^+^ Tregs which were initially elevated showed a progressive return to normal which was adequate since the patient had no evidence of GVHD.

The increased IFN-γ levels, NK cell count and activity correlated with the patient's achievement of molecular disease remission. After one 6-week cycle of rIL-2 treatment the patient's NK cell activity increased from undetectable levels and there was a corresponding increase in IFN-γ levels that remained elevated throughout the treatment course (Figure 2). IFN-γ is a product of activated NK cells which is functionally and integrally linked to NK cytolytic function and also induces expression of the Fas receptor (CD95) on NK targeted cells; thereby, facilitating induction of the extrinsic apoptosis signaling pathway for the target cell [27,28]. One clear-cut mechanism by which tumors can escape immune surveillance and cause leukemic relapse after allo-HCT is associated with dysregulation of pathways that influence immune function by down-regulation of MHC class II genes, which are involved in antigen presentation and this was confirmed by decreased expression of MHC class II at relapse in 17 of 34 patients who had a relapse after transplantation. IFN-γ treatment was shown to rapidly reverse this phenotype in acute myeloid leukemia (AML) blasts of these patients *in vitro* [29].

TKIs may play a role in addressing relapse after HCT either directly by killing leukemic cells or indirectly by stimulating engrafted donor immune effectors. An important consideration is the role of the TKIs in this patient. He had relapsed while on imatinib post-transplant and subsequently while on dasatinib after donor lymphocyte infusion. Registry experiences of the Center for International Blood and Marrow Transplant Research (CIBMTR) and European Group for Blood and Marrow Transplantation (EBMT) evaluating the efficacy of postallo-HCT TKI therapy for Ph^+^ ALL have yielded contrasting results in both leukemia-free survival and overall survival with the CIBMTR reporting no difference in the 3-year cumulative incidence of relapse’ while the EBMT study was associated with a lower relapse rate and improvements in leukemia-free survival [30,31]. Our patient was maintained on TKI during his low-dose rIL-2 immunotherapy treatment with the rationale of maintaining, albeit limited, antileukemic effect while stimulating natural killer function and NKT cells. It is possible that the TKI inhibition of Ph^+^ ALL proliferation contributed to the IL-2 activation of NK cell-mediated clearance of these cells which allowed this patient to achieve undetectable levels of *BCR*-*ABL* expression associated with disease remission.

This report describes a previously unreported approach for treating a multiple relapsed Ph^+^ B-cell ALL-post HCT patient by using a personalized approach of measuring functional NK, quantitative NKT cell, Tregs levels while tailoring the dose and frequency of rIL-2 in response to these effector cells. Our report serves as a basis for low toxicity immunotherapeutic options in patients with poor prognosis disease.

## Conclusion

Multiple relapsed Ph^+^ ALL are associated with a poor prognosis. Our data showed that personalized treatment with low-dose rIL-2 was associated with activation of effector NK cells, which targeted Ph^+^ ALL t (9; 22) and resulted in molecular remission with minimal side effects and maintenance of immune tolerance with Tregs as evidenced by no re-activation of GVHD. We were able to follow the progress and treatment by monitoring the peripheral blood for the NK cell immune activation and by tailoring the treatment dose and frequency to achieve the desired outcome for the patient.

## Future perspective

This case report shows that personalized immunotherapy with rIL-2, in combination with targeted therapy, a TKI, resulted in a complete molecular and cytogenetic remission with minimal adverse events or evidence of GVHD in a patient with relapsed/refractory poor prognosis ALL. This unique approach is an important contribution and supports the need for future research of these combined therapeutic options to improve outcomes and minimize side effects in patients with cancer.

**Executive summary**

- We report a case of complete molecular and cytogenetic remission in relapsed/refractory B-cell acute lymphoblastic leukemia (ALL) following allogeneic hematopoietic stem cell transplantation (allo-HCT) with personalized low-dose immunotherapy treatment using a recombinant human IL-2 (rIL-2), aldesleukin.
- The rIL-2 increased our patient's natural killer cell function ninefold from baseline and natural killer T cells (NKT) levels ninefold following four 4–7 week cycles and – along with a tyrosine kinase inhibitor – resulted in undetectable *BCR-ABL1* p190 transcript levels in peripheral blood and bone marrow.
- The rIL-2 therapy was associated with minimal adverse events and no evidence of graft-versus-host disease.
- Personalized low-dose rIL-2 treatment with dosing and frequency tailored by measurement of a peripheral blood immune panel represents an immunotherapy modality that warrants further investigation given its efficacy and minimal side effects.
